# Sex-Dependent Changes in Gonadotropin-Releasing Hormone Neuron Voltage-Gated Potassium Currents in a Mouse Model of Temporal Lobe Epilepsy

**DOI:** 10.1523/ENEURO.0324-24.2024

**Published:** 2024-10-16

**Authors:** Remya Rajan, Catherine A. Christian-Hinman

**Affiliations:** ^1^Department of Molecular and Integrative Physiology, University of Illinois Urbana-Champaign, Urbana, Illinois 61801; ^2^Neuroscience Program, University of Illinois Urbana-Champaign, Urbana, Illinois 61801; ^3^Beckman Institute for Advanced Science and Technology, University of Illinois Urbana-Champaign, Urbana, Illinois 61801; ^4^Institute for Genomic Biology, University of Illinois Urbana-Champaign, Urbana, Illinois 61801

**Keywords:** GnRH, hypothalamus, kainic acid, seizure, sex differences

## Abstract

Temporal lobe epilepsy (TLE) is the most common focal epilepsy in adults, and people with TLE exhibit higher rates of reproductive endocrine dysfunction. Hypothalamic gonadotropin-releasing hormone (GnRH) neurons regulate reproductive function in mammals by regulating gonadotropin secretion from the anterior pituitary. Previous research demonstrated GnRH neuron hyperexcitability in both sexes in the intrahippocampal kainic acid (IHKA) mouse model of TLE. Fast-inactivating A-type (*I*_A_) and delayed rectifier K-type (*I*_K_) K^+^ currents play critical roles in modulating neuronal excitability, including in GnRH neurons. Here, we tested the hypothesis that GnRH neuron hyperexcitability is associated with reduced *I*_A_ and *I*_K_ conductances. At 2 months after IHKA or control saline injection, when IHKA mice exhibit chronic epilepsy, we recorded GnRH neuron excitability, *I*_A_, and *I*_K_ using whole-cell patch-clamp electrophysiology. GnRH neurons from both IHKA male and diestrus female GnRH-GFP mice exhibited hyperexcitability compared with controls. In IHKA males, although maximum *I*_A_ current density was increased, *I*_K_ recovery from inactivation was significantly slower, consistent with a hyperexcitability phenotype. In IHKA females, however, both *I*_A_ and *I*_K_ were unchanged. Sex differences were not observed in *I*_A_ or *I*_K_ properties in controls, but IHKA mice exhibited sex effects in *I*_A_ properties. These results indicate that although the emergent phenotype of increased GnRH neuron excitability is similar in IHKA males and diestrus females, the underlying mechanisms are distinct. This study thus highlights sex-specific changes in voltage-gated K^+^ currents in GnRH neurons in a mouse model of TLE and suggesting potential sex differences in GnRH neuron ion channel properties.

## Significance Statement

Temporal lobe epilepsy (TLE) is the most common form of focal epilepsy, and people with TLE are at a higher risk of developing reproductive endocrine issues compared with the general population. Previous research found increased excitability of gonadotropin-releasing hormone (GnRH) neurons, which control fertility, in a mouse model of TLE. We investigated whether voltage-gated potassium channels in these neurons play a role in driving this altered excitability by recording the ionic currents from these channels. Although some epilepsy-dependent and sex-specific modifications of potassium conductances were found, the findings overall suggest that epilepsy-associated GnRH neuron hyperexcitability is largely independent of changes in voltage-gated potassium conductances, indicating that other mechanisms are primarily responsible.

## Introduction

Epilepsy is a chronic neurological disorder that affects ∼50 million people worldwide. It is characterized by spontaneous recurring seizures (Z. Chen et al., 2023). People with epilepsy are at higher risk for comorbid disease conditions that complicate its management and increase healthcare costs (Sander, 2013). Temporal lobe epilepsy (TLE) is the most common form of focal epilepsy (Téllez-Zenteno and Hernández-Ronquillo, 2012). People with TLE exhibit a wide range of reproductive endocrine disorders, which have negative impacts on quality of life (Herzog, 2008). For example, in females with TLE, polycystic ovary syndrome (PCOS; Harden, 2005), hypothalamic amenorrhea (Pennell, 2009), functional hyperprolactinemia (Meierkord et al., 1992), and premature menopause (Klein et al., 2001) are exhibited at higher rates than in the general population, and menstrual disorders are observed in one-third of females with epilepsy (Joffe and Hayes, 2008). Similarly, males with epilepsy, particularly TLE, often exhibit hypogonadism, low serum testosterone levels, and hyperprolactinemia (Herzog et al., 1986a; Ziegelbaum, 1991; Bauer et al., 2004; Harden and MacLusky, 2004). Although the use of antiseizure medications (ASMs) can lead to reproductive endocrine dysfunction in patients with TLE (Isojärvi et al., 2005), these comorbidities can also occur in patients not taking ASMs (Herzog et al., 1986a, b; Bilo et al., 1988). Furthermore, the laterality of epileptiform discharges is associated with differential rates of PCOS and hypogonadotropic hypogonadism (HH) in women with TLE (Herzog, 1993). Specifically, PCOS is more common with left-sided TLE, while HH is more common with right-sided TLE, suggesting a direct relationship between seizure activity and downstream reproductive endocrine impacts.

Gonadotropin-releasing hormone (GnRH) neurons in the hypothalamus are the final output in the central control of fertility, and they play an important role in the release of luteinizing hormone (LH) from the anterior pituitary (Constantin, 2011). Altered LH release patterns have been reported in both men and women with epilepsy, suggesting epilepsy-induced changes in GnRH release and GnRH neuronal activity (Herzog et al., 1990; Bilo et al., 1991; Drislane et al., 1994; Quigg et al., 2002). Previous studies found that intrahippocampal kainic acid (IHKA)-injected females that developed prolonged, disrupted estrous cycles exhibited larger changes in basal and mean LH levels from diestrus to estrus compared with control females (Cutia et al., 2023). Furthermore, previous studies also demonstrated changes in GnRH neuron firing activity and intrinsic excitability in the IHKA mouse model of TLE in both males and females (Li et al., 2018; Li and Christian-Hinman, 2022).

Voltage-gated potassium (K^+^) channels are widely expressed in the central and peripheral nervous systems and play a vital role in modulating neuronal excitability and action potentials. Voltage-gated K^+^ channels limit neuronal excitability by mediating outward K^+^ currents, which lead to repolarization and hyperpolarization of the neuronal membrane voltage (Shah and Aizenman, 2014). Therefore, pharmacological or pathophysiological blockade of these K^+^ channels diminishes action potential inhibition and increases neuronal excitability. For example, blocking K^+^ channels with 4-aminopyridine (4-AP) increases neuronal excitability in hippocampal CA1 pyramidal neurons (Jung and Hoffman, 2009). Bath application of 4-AP increased the excitability of rat neocortical pyramidal neurons (Bekkers and Delaney, 2001). Voltage-gated K^+^ (*K*_V_) channels in GnRH neurons exhibit two types of currents, a rapidly inactivating transient A-type current (*I*_A_) and a delayed rectifier (*I*_K_) current. *I*_A_ currents are mediated primarily by K_V_4 channels, whereas *I*_K_ currents are mediated by K_V_2 channels. These channels are also sensitive to modulatory factors that influence GnRH neuron intrinsic excitability. For example, estradiol increases intrinsic excitability in GnRH neurons by reducing *I*_A_ and *I*_K_ conductances (DeFazio and Moenter, 2002), likely through the downregulation of these channels. Similarly, kisspeptin potently increases GnRH neuronal excitability in part by reduction of *I*_A_ conductances (Pielecka-Fortuna et al., 2011). However, whether epilepsy drives changes in voltage-gated potassium conductances in GnRH neurons, thus promoting cellular hyperexcitability, is unknown. Herein, we tested the hypothesis that increased GnRH neuron intrinsic excitability in the IHKA mouse model of TLE is due, at least in part, to reduced voltage-gated *I*_A_ and *I*_K_ currents in both sexes.

## Materials and Methods

### Animals

All experiments and procedures involving animals were conducted in accordance with the guidelines set forth by the Institutional Animal Care and Use Committee of the University of Illinois Urbana-Champaign. Adult GnRH-GFP transgenic mice (033639, The Jackson Laboratory) expressing GFP under the control of GnRH promoter (Suter et al., 2000) were used. The mice were bred and housed under controlled environmental conditions, with a 14:10 h light/dark cycle and room temperature to support regular estrous cyclicity and facilitate breeding (Fox et al., 2006). Continuous access to food and water was provided, and no more than five mice were housed per cage.

### Genotyping

To confirm the presence of GFP transgene, pups were genotyped by standard PCR using the following primers: (1) Transgene Reverse ACA ATC AAG GGT CCC CAA AC; (2) Internal Positive Control Forward AGT GGC CTC TTC CAG AAA TG; (3) Internal Positive Control Reverse TGC GAC TGT GTC TGA TTT CC; and (4) Transgene Forward AAA AGG AAG CTA GGC AGA CAG A (suggested by The Jackson Laboratory).

### Estrous cycle monitoring

As previously described, daily vaginal smears were performed starting at Postnatal Day (P)42 until the day of IHKA injection (Pantier et al., 2019) and extending from 1 month after intrahippocampal saline or kainic acid injections until the day of brain slice preparation. A 15-µl volume of sterile saline was inserted into the vaginal cavity and subsequently collected for the preparation of vaginal smears on a glass slide. The smears were analyzed under a microscope, and the cycle stages were classified as follows: (1) proestrus, dominating nucleated epithelial cells; (2) estrus, dominating cornified epithelial cells; (3) metestrus, presence of both cornified epithelial cells and leukocytes; (4) diestrus, dominating leukocytes; and (5) diestrus few, few leukocytes or no cells. A regular mouse estrous cycle is 4–5 d long (Byers et al., 2012). Mice not exhibiting regular estrous cycles within 3 weeks of preinjection estrous cycle monitoring were not used.

Estrous cycle data collected from 42 d after saline/IHKA injection to the day of slice preparation were used to assess the cycle period phenotype as previously described (Li et al., 2017). Mice exhibiting cycles of 7 d or longer after IHKA injection were categorized as having cycle disruption and named as “IHKA-long.” Conversely, mice that maintained estrous cycle lengths of 4–6 d after IHKA injection were denoted as “IHKA-regular” (Li et al., 2018).

### IHKA/saline injection surgeries

Stereotaxic unilateral injections were performed at ages P56 and older under 2–3% vaporized isoflurane anesthesia (Piramal Critical Care). At the beginning of the surgery, carprofen (0.5 mg/kg, Zoetis) was administered via subcutaneous injection for pain management. Lubricant eye ointment (Alcon Laboratories) was applied to prevent dry eyes. A 50-nl volume of 20 mM KA (Tocris Bioscience or Hello Bio) prepared in 0.9% sterile saline was injected into the right dorsal hippocampus at the following coordinates: 2.0 mm posterior and 1.5 mm lateral to the bregma and 1.4 mm ventral to the cortical surface (Li et al., 2017). The needle was retained at the site of injection for 2 min prior to withdrawal to minimize reflux and ensure proper delivery of KA. Control animals were injected with 50 nl of 0.9% sterile saline. Additional postoperative care included the application of 2.5% lidocaine–2.5% prilocaine cream (Alembic Pharmaceuticals) and Neosporin antibiotic ointment (Johnson and Johnson) to the wound after closing the incision with sutures. At the end of the surgery, 500 µl of 0.9% sterile saline was administered subcutaneously to support hydration.

### Monitoring acute behavioral seizures

Mice were transferred to a warm transparent recovery chamber immediately after surgery. IHKA-injected mice were continuously monitored via video for 3–5 h using Logitech webcams connected to a Windows PC. The recorded videos were thoroughly reviewed for the presence of acute behavioral seizures. Through the video screening, behavioral seizures corresponding to Racine Stage 3 (forelimb clonus) and above (rearing and falling) were noted (Racine, 1972). However, seizures of lower Racine scales could not be documented, due to the limitation of differentiating these seizures from typical mouse behaviors within the video recordings. Furthermore, the videos were also screened for behavior indicative of nonconvulsive status epilepticus, such as freezing or continuous back-circling as previously described for this mouse model of TLE (Bouilleret et al., 1999; Riban et al., 2002).

### Brain slice preparation

Acute brain slices were prepared ∼2 months after IHKA/saline injection. Experiments in female mice were performed on diestrus. As previously described (Li et al., 2018), mice were decapitated, and brains were rapidly excised between zeitgeber time (ZT) 3 and ZT 4, relative to lights-off at ZT 12. The 300-µm-thick coronal brain slices were prepared using a Leica VT1200S (Leica Biosystems) vibrating blade microtome. During slicing, the slices were immersed in an ice-cold, oxygenated (95% O_2_, 5% CO_2_) highly concentrated sucrose solution [containing the following (in mM): 234 sucrose, 11 glucose, 2.5 KCl, 0.5 CaCl_2_, 1.25 NaH_2_PO_4_, and 10 MgSO_4_]. Immediately after slicing, slices were incubated in oxygenated artificial cerebrospinal fluid [ACSF; containing the following (in mM): 126 NaCl, 10 glucose, 2.5 KCl, 0.5 CaCl_2_, 1.25 NaH_2_PO_4_, 1 MgSO_4_, and 26 NaHCO_3_; osmolarity ∼ 298–300 mOsm] at 32°C for 30 min, followed by transfer to room temperature for a minimum of 30 min.

### Electrophysiological recording conditions and data acquisition

The intracellular pipette solution [containing the following (in mM): 125 K-gluconate, 20 KCl, 10 HEPES, 5 EGTA, 0.1 CaCl_2_, 4 MgATP, and 0.4 NaGTP; osmolarity ∼ 305 mOsm], adjusted to a pH of 7.2 using NaOH, was prepared to mimic the native intracellular environment of GnRH neurons (DeFazio and Moenter, 2021). For all K^+^ current recordings, the liquid junction potential of −12.5 mV was calculated using the Clampex 10 software (Molecular Devices) and compensated to ensure accurate membrane potential readings. For all electrophysiological experiments, slices were individually transferred to a recording chamber on the stage of an upright BX51WI microscope (Olympus America). The oxygenated ACSF [osmolarity ∼ 310 mOsm, with synaptic blockers (picrotoxin, DNQX, D-APV) for current-clamp recordings; osmolarity ∼ 310–312 mOsm, with synaptic blockers, cadmium chloride (CdCl_2_), and tetrodotoxin (TTX) for voltage-gated K^+^ current recordings] in the chamber was heated to 30–32°C using an inline heater (Warner Instruments) and circulated at 2.5 ml/min. GnRH neurons expressing GFP were identified using a combination of differential infrared contrast optics using a sCMOS camera (ORCA-Flash 4.0LT, Hamamatsu Photonics) and fluorescence microscopy on a Olympus BX51WI microscope. Data acquisition was conducted using a MultiClamp 700B amplifier, Digidata 1550 digitizer, and Clampex10 software. For current-clamp recordings, the membrane voltage was sampled at 50 kHz and filtered at 10 kHz. Current signals were acquired at 10 kHz and filtered at 4 kHz for all voltage-clamp experiments to ensure signal clarity.

Throughout the course of the experiments, input resistance (*R*_in_), series resistance (*R*_s_), and membrane capacitance were closely monitored. This was achieved by using an access test analyzing the membrane current response to multiple 20 ms, 5 mV hyperpolarizing voltage steps from −65 mV. The access tests were performed before and after each recording to ensure the reliability of measurements throughout the experiments. Cells with *R*_s _< 20 MΩ, *R*_in _> 500 MΩ, and that maintained a stable membrane capacitance (*C*_m_) > 7.5 pF throughout the recordings were used for subsequent analysis. Three cells or fewer were recorded per animal.

### Current-clamp recordings

Whole-cell current–clamp recordings were performed in the presence of 20 µM D-APV, 20 µM DNQX, and 100 µM picrotoxin (Abcam and Hello Bio) to block ionotropic glutamate and GABA receptors. Recording glass pipettes (2–4 MΩ) were filled with the intracellular pipette solution. Once a GΩ seal was obtained, the whole-cell configuration was achieved using conventional techniques. The cell was allowed to stabilize for 2 min prior to the recording. After the stabilization period, an access test was performed. Recordings were initiated after switching the configuration to bridge-balanced current–clamp mode. Cells were maintained at membrane potential of −65 mV by current injection regulated to remain smaller than 100 pA. A series of current steps in 20 pA increments over 1 s, ranging from −5 pA to +95 pA was delivered to test the membrane potential response.

### Voltage-gated K^+^ current recordings

Whole-cell voltage–clamp experiments were performed to record voltage-gated K^+^ currents. These currents were pharmacologically isolated through the blockade of ionotropic GABA and glutamate receptors, as well as fast sodium and calcium channels by adding their respective blockers (20 µM D-APV, 20 µM CNQX, and 100 µM picrotoxin for glutamate and GABA receptors; 1 µM TTX to inhibit sodium channels; and 200 µM CdCl_2_ to inhibit calcium channels). The membrane potential was held at −65 mV between the voltage-clamp protocol sweeps. *R*_s_ was carefully monitored, and only recordings that had *R*_s_ change of <10% during the recording were included in subsequent analysis. The automatic whole-cell compensation feature in the MultiClamp controller software was used immediately before starting the K^+^ current recordings. This procedure enabled precise corrections for *R*_s_ and capacitive transients and removed errors associated with manual compensation methods.

GnRH neurons exhibited two types of voltage-gated K^+^ currents, A-type (*I*_A_) and K-type (*I*_K_) currents. *I*_A_ currents are rapidly inactivating K^+^ currents that are activated at hyperpolarizing membrane potentials, whereas *I*_K_ currents are slowly inactivating currents activated at depolarizing membrane potentials. Both *I*_A_ and *I*_K_ currents were recorded using protocols adapted from previous reports (DeFazio and Moenter, 2002, 2021).

### Characterization of *I*_A_

Our approach to isolating and characterizing *I*_A_ currents was adapted from a previously described protocol (DeFazio and Moenter, 2002), which facilitates the identification of specific prepulse conditions to modulate the inactivation states of these currents. *I*_A_ currents were completely inactivated at −40 mV, whereas a prepulse at −100 mV for 500 ms removed inactivation of these currents.

To determine inactivation and activation of *I*_A_ currents, we used distinct voltage-clamp protocols. For *I*_A_ inactivation, initial hyperpolarization at −110 mV for 100 ms was applied to deinactivate the fast transient *I*_A_ component, followed by a series of prepulse voltage steps of −110 to −10 mV in 10 mV increments for 500 ms and a final test pulse at −10 mV for 500 ms. The fast transient *I*_A_ currents were not seen in response to the −10 mV test pulse after prepulses at values equal to or more depolarized than −40 mV. Therefore, *I*_A_ was isolated by subtracting the current responses for the −10 mV test pulse obtained after the −40 mV prepulse from those elicited at the −10 mV test pulse following the more hyperpolarized prepulses.

To determine the activation of *I*_A_, we applied a prepulse at −100 or −40 mV, followed by test potentials from −100 to +50 mV in 10 mV increments for 500 ms (DeFazio and Moenter, 2021). The *I*_A_ component was isolated by subtracting the currents elicited after the −40 mV prepulse from that after the −100 mV prepulse for each test potential.

To determine the time course of inactivation of *I*_A_, we applied an initial hyperpolarizing membrane potential at −100 mV for 500 ms to remove inactivation, followed by stepping to the inactivation potential at −40 mV for durations of 0, 0.5, 1, 2, 4, 8, 16, 32, 64, 128, 256, 512, or 1,024 ms, with each step succeeded by a test pulse at −10 mV to measure the peak current. To isolate *I*_A_, the peak current obtained at −10 mV test pulse after the 1,024 ms prepulse was subtracted from the peak currents obtained at −10 mV test pulse after the shorter duration prepulses.

Similarly, to characterize the time course of recovery from inactivation of *I*_A_, an initial depolarizing membrane potential at −40 mV for 500 ms was applied to completely inactivate *I*_A_, followed by stepping to −100 mV for the durations listed above, with each step succeeded by a test pulse at −10 mV to measure the peak current. To isolate *I*_A_, the peak current obtained at −10 mV test pulse after the 0 ms prepulse was subtracted from the peak currents obtained at −10 mV test pulse after the subsequent measurements to isolate the transient component.

A P/-8 leak subtraction was employed for all protocols using the built-in feature in the Clampex software. The combination of leak subtraction and whole-cell compensation enabled accurate *I*_A_ measurements and ensured that these measurements were reflective of the currents under physiological conditions.

### Characterization of *I*_K_

Due to the slow inactivation kinetics of *I*_K_ currents, a separate set of recording protocols was used to investigate *I*_K_ currents as outlined previously, facilitating the use of more moderate command potentials and durations that were compatible with the physiological tolerance of the cells (DeFazio and Moenter, 2021).

To determine the voltage dependence of activation of *I*_K_, we used an initial prepulse at −75 mV for 10 s to remove inactivation of *I*_K_. A second prepulse at −50 mV was applied for 1 s to drive complete inactivation of *I*_A_. The peak *I*_K_ current was then measured at test pulses ranging from −50 to +50 mV in 10 mV increments for 10 s. The inactivation of *I*_K_ was measured in the same protocol by applying a final step to +50 mV for 100 ms.

The time course of inactivation of *I*_K_ was characterized by an initial prepulse at −75 mV for 10 s to remove inactivation of *I*_K_, followed by a second prepulse at −50 mV for 1 s for complete inactivation of *I*_A_. This was followed by inactivation pulse at −30 mV for durations of 0, 0.1, 0.2, 0.4, 0.8, 1.6, 3.2, 6.4, 12.8, 25.6, and 51.2 s, with each step succeeded by a test pulse at +50 mV to measure the peak current. To characterize the time course of recovery from inactivation of *I*_K_, an initial prepulse at +50 mV for 10 s was used to inactivate *I*_K_ followed by a series of recovery pulses at −80 mV for durations 0, 0.1, 0.2, 0.4, 0.8, 1.6, 3.2, 6.4, 12.8, 25.6, and 51.2 s. A brief pulse at −50 mV for 1 s was used to inactivate *I*_A,_ followed by a test pulse at +50 mV for 100 ms to measure the peak *I*_K_ current.

Due to the specific experimental conditions and durations required for recording *I*_K_ currents, leak subtraction could not be performed. However, to ensure the accuracy of the recordings, we implemented automatic whole-cell compensation to correct for whole-cell capacitance and *R*_s_ artifacts.

### Histology and staining

After collecting the slices containing the hypothalamus for electrophysiology, the remaining portion of the brain was fixed in 4% paraformaldehyde at 4°C for 24 h. It was then transferred to 30% sucrose solution with 0.5% sodium azide at 4°C until sectioning. Coronal hippocampal sections of 40 µm thickness were made from the dorsal hippocampal region using a freezing microtome (SM2010R, Leica Biosystems). Eight to twelve sections per mouse were used for assessment of hippocampal sclerosis via cresyl violet staining and/or gliosis via GFAP (glial fibrillary acidic protein) staining. For cresyl violet staining, sections were mounted on charged glass slides and stained with cresyl violet (Sigma-Aldrich C5042) for 12 min at room temperature. Sections were then dehydrated through graded ethanol solutions (70–100%) and cleared in xylene. For GFAP immunostaining, free-floating sections were incubated in anti-GFAP mouse monoclonal antibody (1:1,000, Sigma-Aldrich G3893) for 48 h at 4°C on a shaker, followed by incubation in fluorescein horse anti-mouse secondary antibody (1:1,000, Vector Laboratories FI-2000) for 2 h at room temperature. The sections were then mounted on a glass slide, covered with coverslips, and then imaged using a BX43 light and fluorescence microscope (Olympus Life Science), with an Infinity3 camera and Infinity Analyze software (Teledyne Lumenera).

### Data analysis and statistics

Data analysis was performed using Clampfit 11.2 and Python 3.12.0 for all parameters. For intrinsic excitability analysis, the area under the frequency-injected current (*F*–*I*) curve for each cell was calculated using a trapezoidal method. For K^+^ current recordings, the current density was calculated by dividing the peak current by cell capacitance. To calculate the voltage dependence of activation of *I*_A_ and *I*_K_, the peak current was normalized, divided by the driving force calculated using the Goldman–Hodgkin–Katz current equation (K^+^ reversal potential, −106.77 mV; Clay, 2009, 2000), plotted as a function of command potential and subsequently fit with the Boltzmann equation as follows:

$$y=A_{1}+\;\frac{\left(A_{1}-A_{2}\right)}{1+e^{\frac{\left(V_{1/2}-V\right)}{k}}},$$

where *y* is the value at a given command potential *V*, *A*_1_ is the initial value, *A*_2_ is the final value, *V*_1/2_ is the half-maximal voltage, *V* is the command potential, and *k* is the slope factor. The inactivation curves were also fit to the same equation to calculate the *V*_1/2_ and *k* of inactivation of *I*_A_ and *I*_K_.

For statistical analysis, OriginPro 10.1 and Python 3.12.0 were used. To compare passive properties (*R*_in_, *R*_s_, and cell capacitance), we used independent two-sample *t* tests for data from male groups, and one-way ANOVAs were used to evaluate differences among female groups. To evaluate neuronal excitability, a two-way ANOVA was used to evaluate the effects of sex and treatment on the evoked firing rate, with IHKA-regular and IHKA-long females combined. Separate comparisons between saline and IHKA male groups were done using an independent two-sample *t* test, and a one-way ANOVA was conducted for comparisons within the three female groups. Prior to the ANOVA, the normality of the residuals was assessed using the Shapiro–Wilk test, and the homogeneity of variances was tested using Levene's test. For voltage and time dependence of inactivation and activation, comparisons within sexes were conducted using two-way repeated–measure ANOVAs. Mauchly's test of sphericity was conducted to assess the assumption of sphericity in the repeated-measure ANOVA, and where the assumption was violated, Greenhouse–Geisser correction was applied to adjust the degrees of freedom. For the Boltzmann fit parameters, two-way ANOVAs were utilized to analyze the differences. Post hoc comparisons using Tukey’s HSD tests were done wherever the ANOVA results showed a significant difference.

## Results

### Confirmation of targeted IHKA injection

As previously described (Li et al., 2018), all IHKA male and female mice were screened for two or more acute behavioral seizures immediately following the IHKA injection via video recording for 3–5 h. Of the 56 IHKA-injected mice (24 males, 32 females), 48 mice displayed two or more acute behavioral seizures during this time. All mice that did not show two or more acute behavioral seizures were evaluated for hippocampal sclerosis via cresyl violet (Nissl) staining at ∼2 months after injection. Of these eight mice, all showed hippocampal sclerosis, which confirmed the targeted IHKA injections. Therefore, all 56 IHKA-injected mice were used in the study. Of note, previous studies involving a cohort of identically IHKA-injected mice showed that spontaneous recurrent seizures in hippocampal depth electroencephalography recordings were displayed in all mice within 1 month postinjection (Cutia et al., 2022). This successful high rate of epilepsy induction in this IHKA mouse model supports the likelihood that the 56 KA-injected mice in the current study also developed chronic epilepsy within 2 months postinjection.

### Increased intrinsic excitability in GnRH neurons from IHKA GnRH-GFP mice

**Figure 1.**
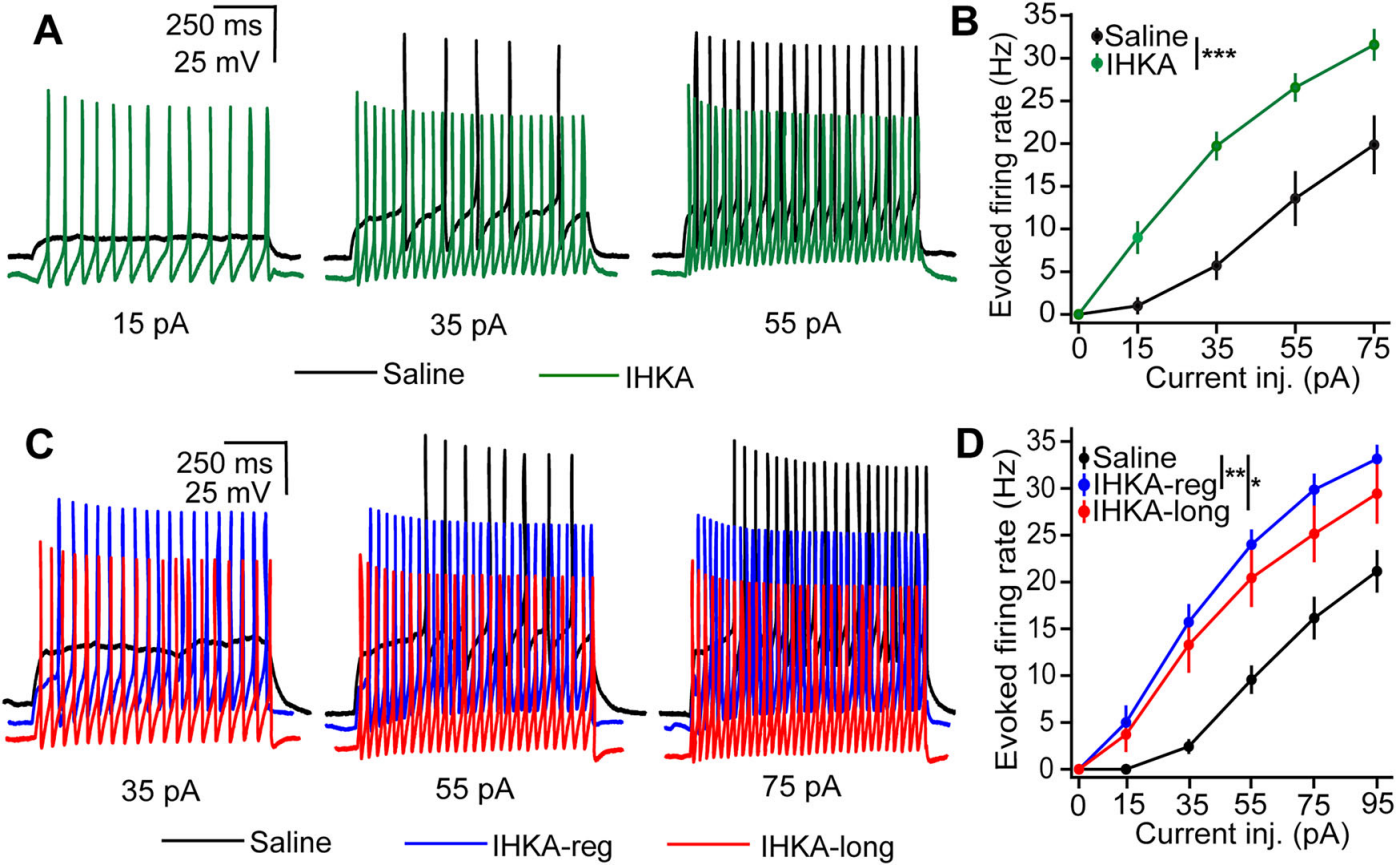
Impact of IHKA injection on GnRH neuron intrinsic excitability in GnRH-GFP male and female mice. ***A***, Representative examples of evoked firing in response to depolarizing current steps in cells from saline (black) and IHKA (green)-treated males. Note that the IHKA traces are the offset to show differences in spiking and do not show hyperpolarization of the resting membrane potential. ***B***, Frequency–current (*F*–*I*) curves for GnRH neurons recorded from saline (black; *n* = 7 cells, 5 mice) and IHKA (green; *n* = 7 cells, 5 mice) males; ***p < 0.001 for comparisons of the area under the curve (AUC) by independent two-sample *t* test. ***C***, Representative examples of evoked firing in response to depolarizing current steps in cells from saline (black) and IHKA-regular (blue) and IHKA-long (red) females. The IHKA-regular and IHKA-long traces are the offset to show differences in spiking. ***D***, *F*–*I* curves for GnRH neurons recorded from saline (*n* = 7 cells, 5 mice) and IHKA-regular (*n* = 7 cells, 5 mice) and IHKA-long females (*n* = 7 cells, 5 mice); **p < 0.01 and *p < 0.05 for comparisons of AUC by one-way ANOVA with Tukey’s HSD test.

Previous studies in which GnRH neurons were visualized using a tdTomato Cre-reporter strategy demonstrated increased intrinsic excitability in GnRH neurons from both male and female IHKA mice (Li et al., 2018). Here, we examined if this increase in intrinsic excitability is also seen in GnRH neurons from GnRH-GFP transgenic mice in current-clamp recordings. All recordings from female mice were performed on diestrus. Two-way ANOVA showed that sex and treatment had significant effects on the evoked firing rate in GnRH neurons, with treatment having a more substantial impact (*p* = <0.0001). Overall, males showed higher excitability when compared with females (*p* = 0.034). However, there was no interaction effect between sex and treatment, suggesting that the effect of treatment on the evoked firing rate did not differ between males and females (Table 1). An independent two-sample *t* test showed a significant increase in the evoked firing rate in IHKA males when compared with their saline-injected counterparts (*p* = 0.00048). Comparisons between saline, IKHA-regular, and IHKA-long females were done using one-way ANOVA followed by Tukey's HSD. Both IHKA-regular and IHKA-long groups showed increased firing rates compared with saline controls (saline vs IHKA-regular, *p* = 0.001; saline vs IHKA-long, *p* = 0.011). However, there was no difference in firing rates between IHKA-regular and IHKA-long groups (*p* = 0.548; Fig. 1*A*–*D*). Overall, these results demonstrate increased GnRH neuron excitability in IHKA-injected GnRH-GFP males and females when compared with their saline counterparts, confirming and extending previous findings. There were no differences in the *R*_in_ (saline, 617.40 ± 38.2 MΩ; IHKA, 920.90 ± 138.6 MΩ; *n* = 7 cells, 5 mice per group; *p* = 0.563 calculated by independent two-sample *t* test) or capacitance (saline, 14.67 ± 1.4 pF; IHKA, 14.65 ± 1.5 pF; *n* = 7 cells, 5 mice per group; *p* = 0.990, independent two-sample *t* test) between saline and IHKA males. Similarly, there were no differences in *R*_in_ (saline, 766.46 ± 110.2 MΩ; IHKA-regular, 768.83 ± 83.1 MΩ; IHKA-long, 766.57 ± 60.5 MΩ; *n* = 7 cells, 5 mice per group; *p* = 0.999, one-way ANOVA) or capacitance (saline, 16.23 ± 2.9 pF; IHKA-regular, 11.45 ± 1.4 pF; IHKA-long, 15.72 ± 1.1 pF; *n* = 7 cells, 5 mice per group; *p* = 0.192, one-way ANOVA) between saline, IHKA-regular, and IHKA-long females.

**Table 1.** Two-way ANOVA statistical parameters for action potential firing using AUC calculated by the trapezoidal method

Overall ANOVA						
	*F*	*p* value				
Sex	(1, 31) 4.933	**0.034**				
Treatment	(1, 31) 40.994	**<0**.**0001**				
Sex: Treatment	(1, 31) 0.239	0.628				
Overall	(3, 31) 14.260	**<0**.**0001**				

Tukey’s HSD						
Sex	Treatment	Sex	Treatment	Diff	*p* value	LCL—UCL
Male	IHKA	Male	Saline	797.071	**0**.**0004**	322.479—1271.663
Female	Saline	Male	Saline	−200.357	0.66457	−674.949—274.235
Female	IHKA	Male	IHKA	−313.429	0.18542	−724.437—97.580
Female	IHKA	Female	Saline	684	**0**.**0005**	272.991—1095.009

Parameters shown in bold showed significant differences. Note that the data from IHKA-regular and IHKA-long females are combined.

### In IHKA male mice, GnRH neuron A-type K^+^ current density is increased but other current properties are unchanged

We hypothesized that altered voltage-gated K^+^ currents are responsible, at least in part, for the observed increase in GnRH neuron excitability. Therefore, we used whole-cell voltage–clamp recordings to determine if voltage-gated K^+^ currents in GnRH neurons are altered in IHKA mice, focusing on fast-inactivating *I*_A_ currents and slowly inactivating *I*_K_ currents.

**Figure 2.**
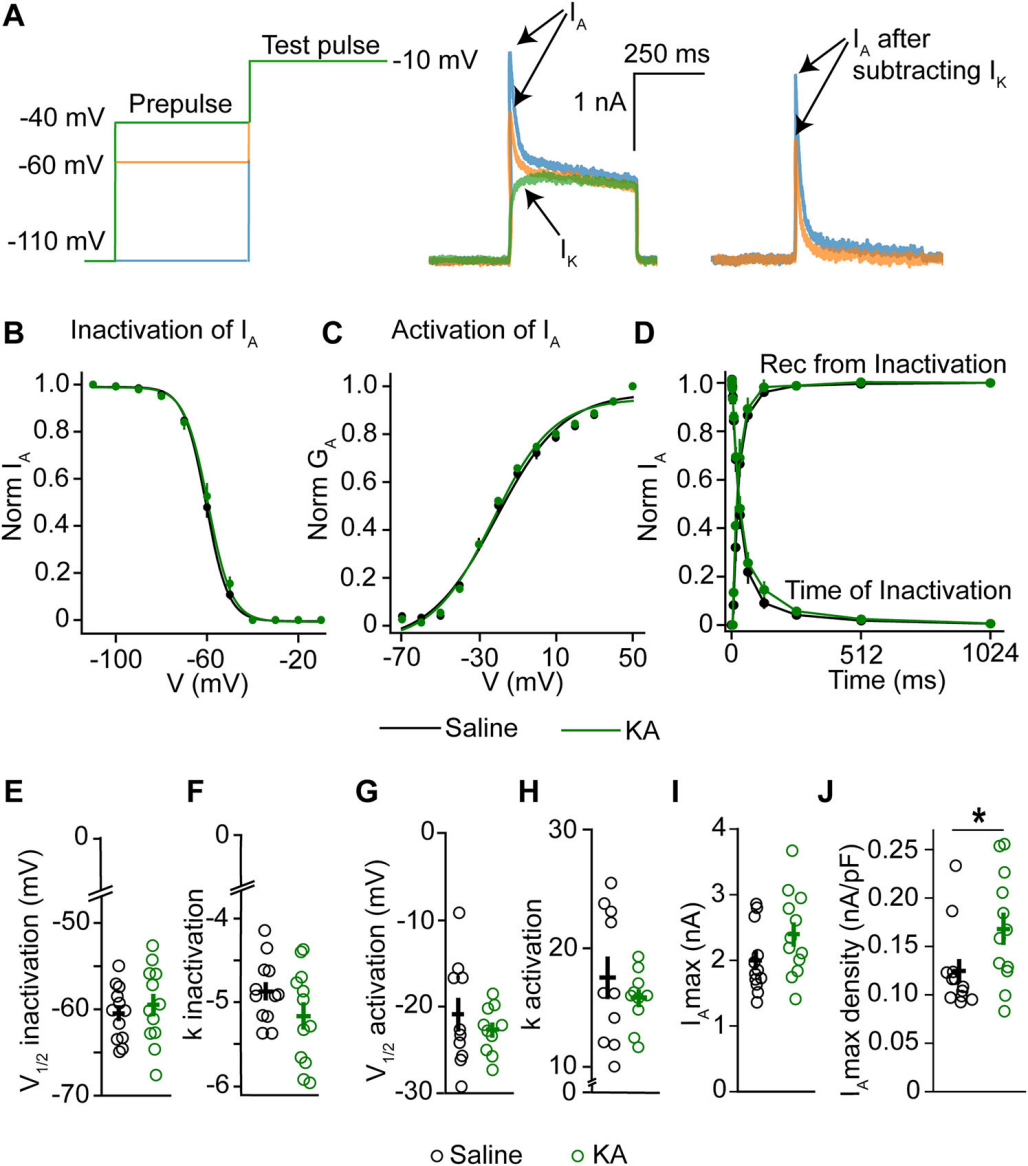
Characterization of *I*_A_ potassium current in GnRH-GFP male mice. ***A***, Voltage-clamp protocol used for characterizing the voltage dependence of inactivation of *I*_A_ currents; only three prepulse potentials are shown for clarity (left); representative trace of K^+^ currents in GnRH neurons from saline-treated male mice illustrating *I*_A_ and *I*_K_ currents (middle); representative traces showing *I*_A_ after subtracting *I*_K_ (right). ***B***, Voltage dependence of inactivation, normalized by the maximum current; saline (*n* = 12 cells, 8 mice), IHKA (*n* = 12 cells, 8 mice). ***C***, Voltage dependence of activation, normalized by the maximum conductance; saline (*n* = 10 cells, 8 mice), IHKA (*n* = 10 cells, 8 mice). ***D***, Time course of recovery from inactivation and time course of inactivation, normalized by the maximum current; saline (*n* = 12 cells, 8 mice), IHKA (*n* = 12 cells, 8 mice). ***E***, *V*_1/2_ inactivation; membrane potential at which half of the current is inactivated. ***F***, *k* inactivation slope factor. ***G***, *V*_1/2_ activation; membrane potential at which half of the current is activated. ***H***, *k* activation slope factor. ***I***, Maximum current. ***J***, Maximum current density; *p < 0.05 using an independent two-sample *t* test.

To isolate and characterize *I*_A_, the *I*_K_ component of the K^+^ current was subtracted from the total current (Fig. 2*A*). In males, the voltage dependence of inactivation and activation of *I*_A_ in GnRH neurons did not change in IHKA mice, as tested in two-way repeated–measure ANOVA (inactivation, *p* = 0.645; activation, *p* = 0.873; Fig. 2*B*,*C*; Table 2). IHKA treatment also did not alter the time dependence of *I*_A_. Two-way repeated–measure ANOVA showed no significant difference in the time course of recovery from inactivation between saline and IHKA males (*p* = 0.521; Fig. 2*D*; Table 2). The time course of inactivation was also not altered in IHKA mice (*p* = 0.523; Fig. 2*D*; Table 2). The *V*_1/2_ and slope factor of inactivation and activation of *I*_A_ remained unchanged in IHKA mice (*V*_1/2_ inactivation, *p* = 0.566; *k* inactivation, *p* = 0.150; *V*_1/2_ activation, *p* = 0.244; *k* activation, *p* = 0.712; calculated by independent two-sample *t* test; Fig. 2*E*–*H*). Similarly, the maximum *I*_A_ current was also not changed (*p* = 0.108; calculated by independent two-sample *t* test; Fig. 2*I*). However, the maximum *I*_A_ current density was increased in IHKA mice (*p* = 0.047; calculated by independent two-sample *t* test; Fig. 2*J*), while cell capacitance remained unchanged (saline, 16.41 ± 0.7 pF; IHKA, 15.39 ± 1.4 pF; *n* = 12 cells, 5 mice per group; *p* = 0.546; independent two-sample *t* test).

**Table 2.** Two-way repeated–measure ANOVA statistical parameters for characterizing the effect of saline/IHKA treatment on voltage and time dependence of *I*_A_ and *I*_K_ in male mice

	Assumption of sphericity met?	Correction used?	Effect of treatment	Significant?
*I*_A_				
Inactivation	**X**	✔	*F*_(1, 22)_ = 0.218, *p* = 0.645	No
Activation	**X**	✔	*F*_(1, 22)_ = 0.026, *p* = 0.873	No
Recovery from inactivation	**X**	✔	*F*_(1, 22)_ = 0.425, *p* = 0.521	No
Time course of inactivation	✔	**X**	*F*_(1, 21)_ = 0.521, *p* = 0.523	No
*I*_K_				
Inactivation	**X**	✔	*F*_(1, 21)_ = 0.073, *p* = 0.790	No
Activation	**X**	✔	*F*_(1, 21)_ = 0.007, *p* = 0.934	No
**Recovery from inactivation**	**X**	✔	***F*_(1, 18)_ = 9.383, *p* = 0.007**	**Yes**
Time course of inactivation	**X**	✔	*F*_(1, 22)_ = 0.102, *p* = 0.753	No
Tukey’s HSD for comparing recovery from inactivation of *I*_K_ currents				
Time (ms)	Mean diff	95% CI	*p* value	Significant?
0	0.064	−0.017–0.144	0.119	No
**0.1**	**0**.**111**	**0.030–0.191**	**0.007**	**Yes**
**0.2**	**0**.**140**	**0.059–0.219**	**0.0007**	**Yes**
**0.4**	**0**.**158**	**0.078–0.238**	**0.0001**	**Yes**
**0.8**	**0**.**155**	**0.075–0.235**	**0.0002**	**Yes**
**1.6**	**0**.**146**	**0.065–0.226**	**0.0004**	**Yes**
**3.2**	**0**.**125**	**0.045–0.206**	**0.002**	**Yes**
**6.4**	**0**.**099**	**0.020–0.180**	**0.015**	**Yes**
12.8	0.068	−0.012–0.148	0.095	No
25.6	0.023	−0.057–0.103	0.577	No
51.2	<0.00001	−0.080–0.080	1	No

In the upper panel, the outcomes of two-way repeated–measure ANOVA tests are given. The analysis was conducted with treatment as one variable and repeated measures on command potential or time interval as the second variable. Tests for assumption of sphericity and correction used are also indicated. Parameters shown in bold showed significant differences. In the lower panel, Tukey’s HSD post hoc test results are provided for the recovery from inactivation of *I*_K_ currents at different time points. The mean differences, 95% confidence intervals (CI), *p* values, and significance (*p* < 0.05) are reported, highlighting the time points where recovery from inactivation showed significant differences between treatments. Parameters shown in bold showed significant differences.

### Slower time course of recovery from inactivation of K-type K^+^ currents in GnRH neurons in IHKA male mice

**Figure 3.**
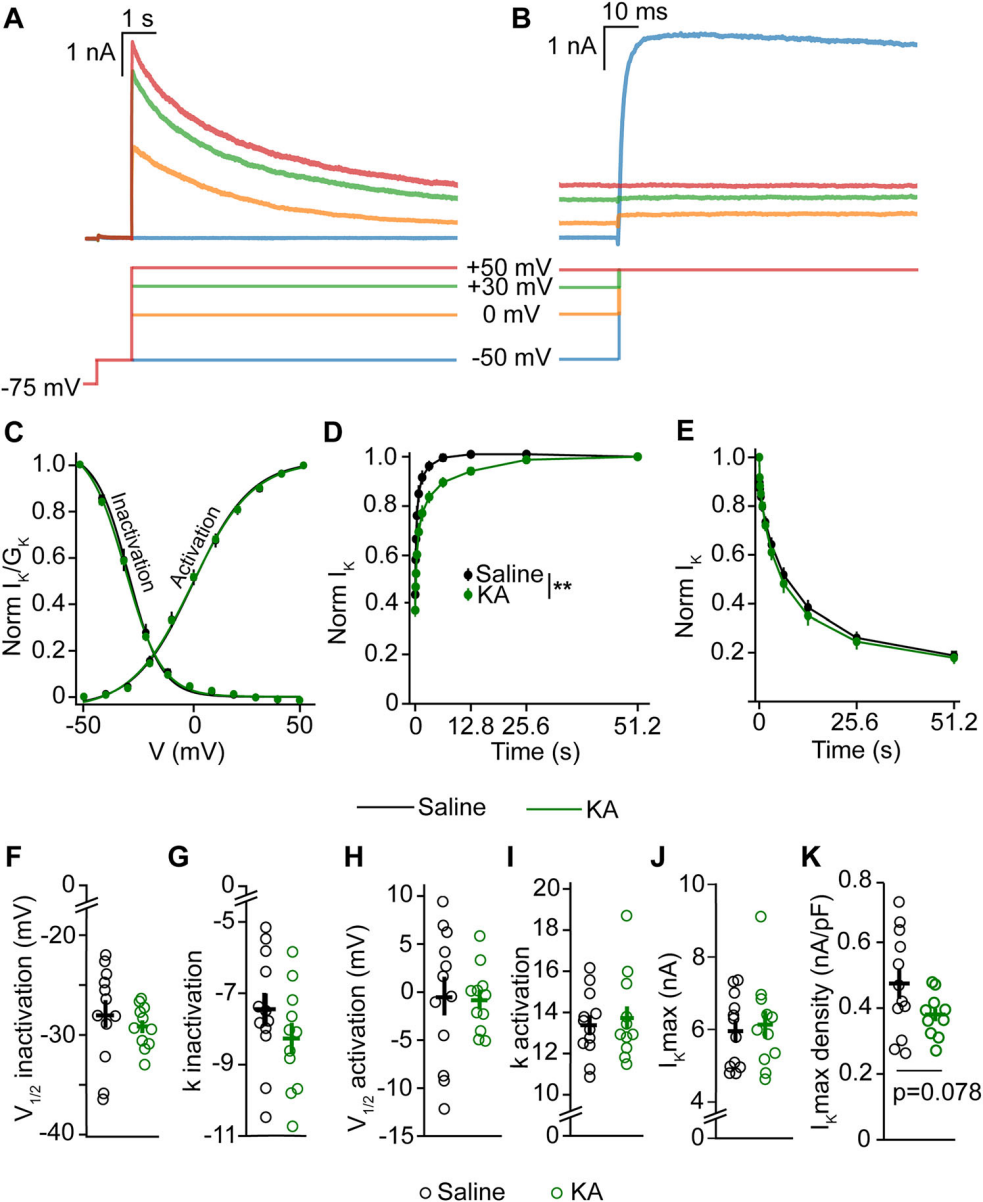
Characterization of *I*_K_ potassium current in GnRH-GFP male mice. ***A***, Representative traces illustrating the activation of *I*_K_ (top) and the voltage protocols used (bottom). ***B***, Representative traces illustrating the inactivation of *I*_K_ (top) and the voltage protocols used (bottom). ***C***, Voltage dependence of inactivation and activation, normalized by the maximum current and maximum conductance, respectively; saline (*n* = 12 cells, 8 mice), IHKA (*n* = 11 cells, 8 mice). ***D***, Time course of recovery from inactivation; saline (*n* = 10 cells, 6 mice), IHKA (*n* = 12 cells, 6 mice); **p < 0.01 using two-way repeated–measure ANOVA with Tukey’s HSD test. ***E***, Time course of inactivation; saline (*n* = 12 cells, 8 mice), IHKA (*n* = 12 cells, 8 mice). ***F–I***, (***F***); *V*_1/2_ inactivation (***G***); *k* inactivation slope factor (***H***); *V*_1/2_ activation (***I***); *k* activation slope factor (***J***); maximum current. (***K***); maximum current density.

With respect to the voltage dependence of *I*_K_, two-way repeated–measure ANOVA showed no differences between saline and IHKA males in the voltage dependence of inactivation (*p* = 0.790) or activation (*p* = 0.934; Fig. 3*A*–*C*; Table 2).

The time course of recovery from inactivation, however, did exhibit a difference between saline and IHKA males, as shown in two-way repeated–measure ANOVA (*p* = 0.007; Fig. 3*D*; Table 2). Comparisons at individual time points were done using Tukey’s HSD post hoc tests showed significant differences at 0.1 s (*p* = 0.007), 0.2 s (*p* = 0.001), 0.4 s (*p* = 0.0001), 0.8 s (*p* = 0.0002), 1.6 s (0.0004), 3.2 s (*p* = 0.002), and 6.4 s intervals (*p* = 0.015). By contrast, there was no significant change in the time course of inactivation upon IHKA treatment (*p* = 0.753; Fig. 3*E*; Table 2). These results indicate that the time course of *I*_K_ recovery from inactivation is slower in IHKA males compared with controls, consistent with a phenotype of increased excitability. No significant differences were seen in the *V*_1/2_ and slope factor of inactivation and activation of *I*_K_ (*V*_1/2_ inactivation, *p* = 0.425; *k* inactivation, *p* = 0.226; *V*_1/2_ activation, *p* = 0.871; *k* activation, *p* = 0.646; calculated by independent two-sample *t* test; Fig. 3*F*–*I*). Similarly, the maximum *I*_K_ current and maximum *I*_K_ current density were also not changed in IHKA mice (maximum *I*_K_ current, *p* = 0.697; maximum *I*_K_ current density, *p* = 0.078; calculated by independent two-sample *t* test; Fig. 3*J*,*K*).

### Voltage-gated K^+^ currents in GnRH neurons are largely unchanged in IHKA female mice

**Figure 4.**
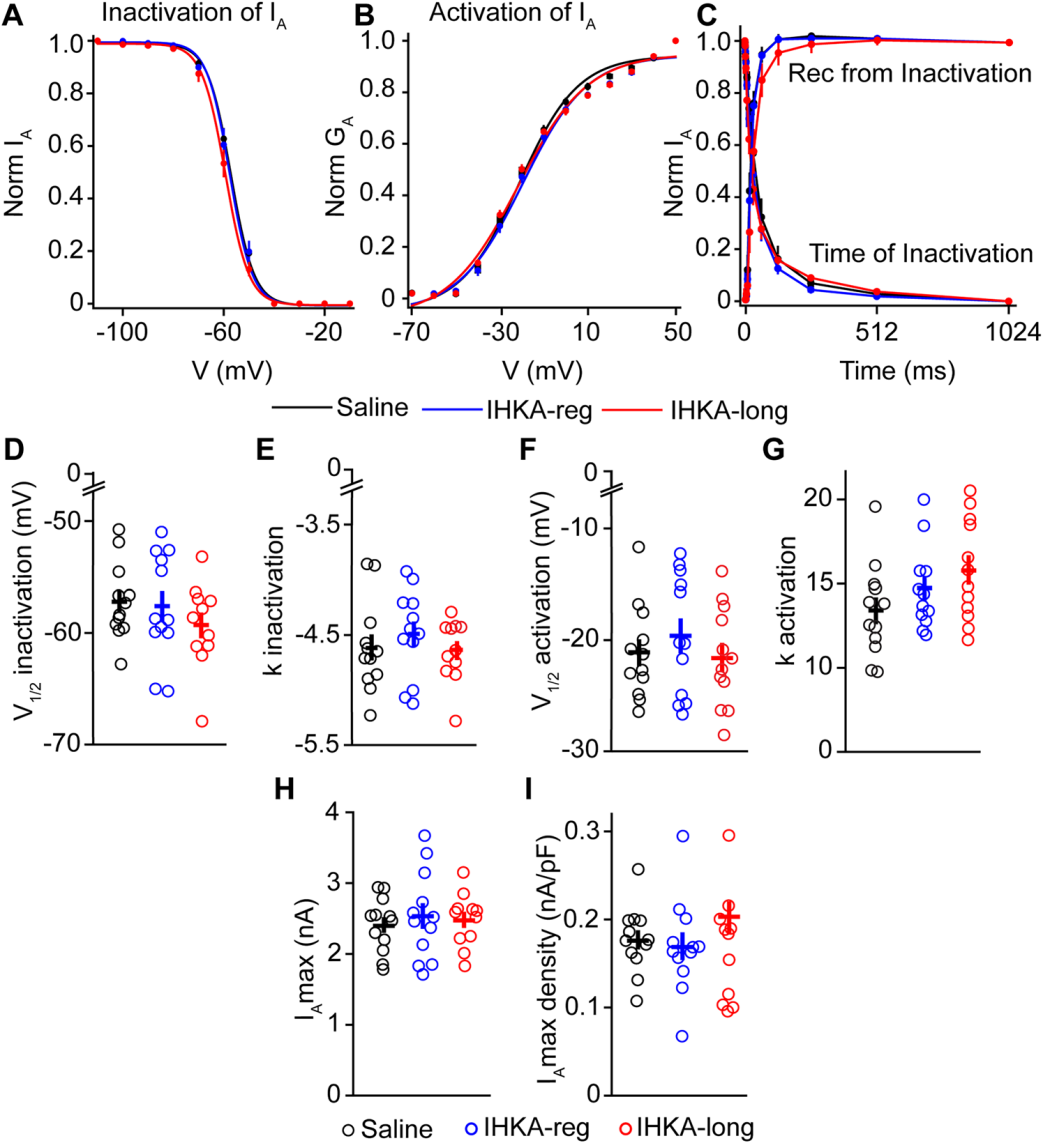
Characterization of *I*_A_ potassium current in GnRH-GFP female mice. ***A***, Voltage dependence of inactivation, normalized by the maximum current; saline (*n* = 12 cells, 8 mice), IHKA-regular (*n* = 12 cells, 8 mice), IHKA-long (*n* = 11 cells, 8 mice). ***B***, Voltage dependence of activation, normalized by maximum conductance; saline (*n* = 12 cells, 7 mice), IHKA-regular (*n* = 12 cells, 8 mice), IHKA-long (*n* = 12 cells, 8 mice). ***C***, Time course of recovery from inactivation; saline (*n* = 10 cells, 7 mice), IHKA-regular (*n* = 12 cells, 8 mice), IHKA-long (*n* = 12 cells, 7 mice), and time course of inactivation; saline (*n* = 10 cells, 7 mice), IHKA-regular (*n* = 12 cells, 8 mice), IHKA-long (*n* = 12 cells, 8 mice), normalized by the maximum current. ***D***, *V*_1/2_ inactivation. ***E***, *k* inactivation slope factor. ***F***, *V*_1/2_ activation. ***G***, *k* activation slope factor. ***H***, Maximum current. ***I***, Maximum current density.

In IHKA diestrus females, the voltage and time dependence of *I*_A_ currents (inactivation, *p* = 0.325; activation, *p* = 0.619; time course of recovery, *p* = 0.218; time course of inactivation, *p* = 0.479; calculated by two-way repeated–measure ANOVA; Fig. 4*A*–*C*) did not show any changes (Table 3). The *V*_1/2_ and slope factor of inactivation and activation of *I*_A_ were also unchanged in IHKA females (*V*_1/2_ inactivation, *p* = 0.422; *k* inactivation, *p* = 0.592; *V*_1/2_ activation, *p* = 0.579; *k* activation, *p* = 0.116; calculated by one-way ANOVA; Fig. 4*D*–*G*). Similarly, the maximum *I*_A_ current and the maximum *I*_A_ current density were also not altered (maximum *I*_A_ current, *p* = 0.812; maximum *I*_A_ current density, *p* = 0.309; calculated by one-way ANOVA; Fig. 4*H*,*I*).

**Table 3.** Two-way repeated–measure ANOVA statistical parameters for characterizing the effect of saline/IHKA treatment on voltage and time dependence of *I*_A_ and *I*_K_ in female mice

	Assumption of sphericity met?	Correction used?	Effect of treatment	Significant?
*I*_A_				
Inactivation	✔	**X**	*F*_(2, 32)_ = 0.998, *p* = 0.325	No
Activation	**X**	✔	*F*_(2, 33)_ = 0.486, *p* = 0.619	No
Recovery from inactivation	**X**	✔	*F*_(2, 30)_ = 1.579, *p* = 0.218	No
Time course of inactivation	**X**	✔	*F*_(2, 31)_ = 0.513, *p* = 0.479	No
*I*_K_				
Inactivation	**X**	✔	*F*_(2, 26)_ = 0.192, *p* = 0.665	No
Activation	**X**	✔	*F*_(2, 26)_ = 0.166, *p* = 0.687	No
Recovery from inactivation	**X**	✔	*F*_(2, 16)_ = 0.387, *p* = 0.543	No
Time course of inactivation	**X**	✔	*F*_(2, 21)_ = 0.137, *p* = 0.714	No

The outcomes of two-way repeated–measure ANOVA tests are given. The analysis was conducted with treatment as one variable and repeated measures on command potential or time interval as the second variable. Tests for assumption of sphericity and correction used are also indicated.

**Figure 5.**
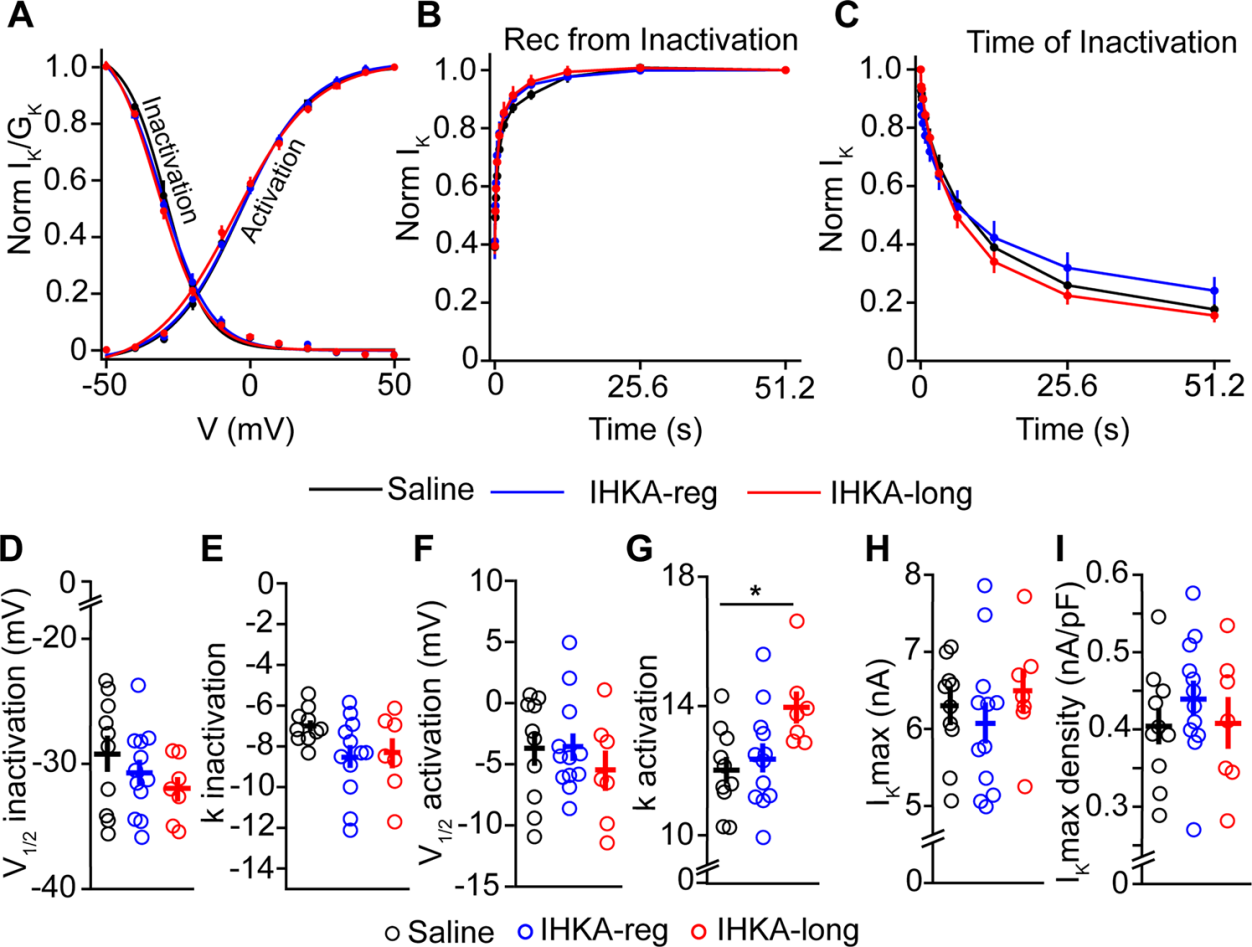
Characterization of *I*_K_ potassium current in GnRH-GFP female mice. ***A***, Voltage dependence of inactivation and activation, normalized by the maximum current and maximum conductance, respectively; saline (*n* = 10 cells, 5 mice), IHKA-regular (*n* = 12 cells, 7 mice), IHKA-long (*n* = 7 cells, 4 mice). ***B***, Time course of recovery from inactivation; saline (*n* = 6 cells, 3 mice), IHKA-regular (*n* = 6 cells, 5 mice), IHKA-long (*n* = 7 cells, 4 mice). ***C***, Time course of inactivation; saline (*n* = 7 cells, 3 mice), IHKA-regular (*n* = 10 cells, 5 mice), IHKA-long (*n* = 7 cells, 3 mice). ***D***, *V*_1/2_ inactivation. ***E***, *k* inactivation slope factor. ***F***, *V*_1/2_ activation. ***G***, *k* activation slope factor; *p < 0.05 using one-way ANOVA with Tukey’s HSD test. ***H***, Maximum current. ***I***, Maximum current density.

With respect to *I*_K_, there was no change in the voltage and time dependence of these currents (inactivation, *p* = 0.665; activation, *p* = 0.687; time course of recovery, *p* = 0.543; time course of inactivation, *p* = 0.714; calculated by two-way repeated–measure ANOVA; Fig. 5*A*–*C*; Table 3). Other parameters such as *V*_1/2_ inactivation (*p* = 0.307), inactivation slope factor (*p* = 0.095), *V*_1/2_ activation (*p* = 0.590), maximum *I*_K_ current (*p* = 0.582), and maximum *I*_K_ current density (*p* = 0.532) also didn't show any differences. However, the activation slope factor did exhibit a significant difference (*p* = 0.027; calculated by one-way ANOVA; Fig. 5*D*–*I*).

### Effects of sex and IHKA status on biophysical properties of *I*_A_ and *I*_K_

**Figure 6.**
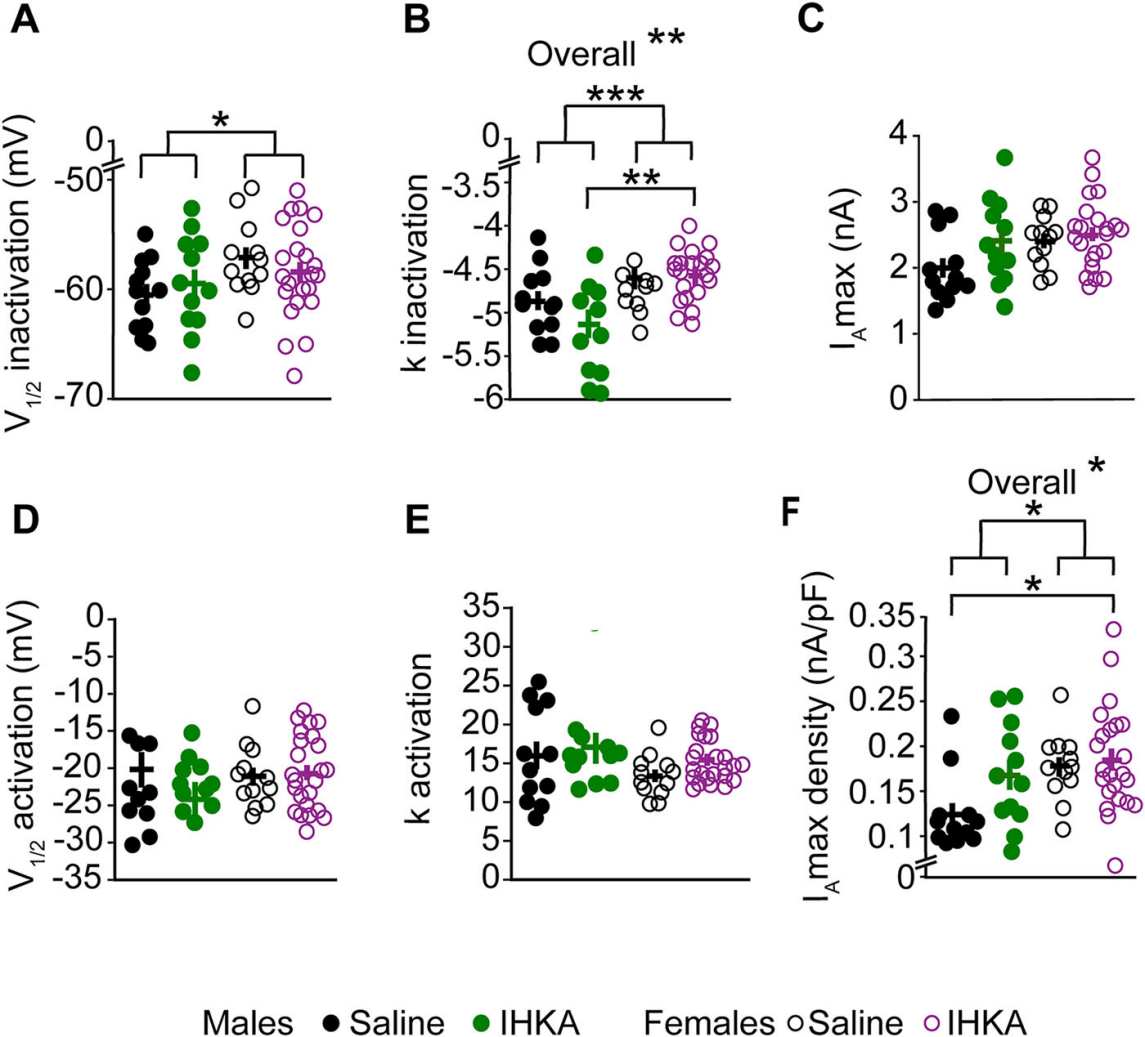
Effects of sex and IHKA status on the properties of *I*_A_. ***A***, *V*_1/2_ inactivation; *p < 0.05 using two-way ANOVA. ***B***, *k* inactivation slope factor; **p < 0.01; ***p < 0.001 using two-way ANOVA with Tukey’s HSD test. ***C***, Maximum current. ***D***, *V*_1/2_ activation. ***E***, *k* activation slope factor. ***F***, Maximum current density; *p < 0.05 using two-way ANOVA with Tukey’s HSD test. Note that data from IHKA-regular and IHKA-long female mice are combined.

As there were no differences in the voltage dependence of inactivation and activation of *I*_A_ or *I*_K_ in IHKA-regular and IHKA-long female mice, the data from these groups were combined to determine the effects of sex, treatment, or their interaction on K^+^ current properties. Two-way ANOVA revealed differences based on sex for *V*_1/2_ inactivation (*p* = 0.049), the inactivation slope factor (*p* = 0.0005), and maximum current density (*p* = 0.017) for *I*_A_ (Fig. 6). Post hoc Tukey’s HSD tests revealed differences in the inactivation slope factor of *I*_A_ between IHKA males and IHKA females (*p* = 0.001), with faster inactivation for IHKA males than IHKA females. Although an effect of treatment and the interaction between sex and treatment were not significant for most properties, the overall models for the inactivation slope factor (*p* = 0.001) and *I*_A_ maximum current density (*p* = 0.016) were significant, suggesting that the combination of sex, treatment, and their interaction explains a major portion of the variance in these properties. By contrast, *V*_1/2_ activation and the activation slope factor did not show differences based on sex, treatment, or interaction. These findings highlight the importance of sex as a factor influencing specific ion channel properties in GnRH neurons, particularly inactivation characteristics of *I*_A_ and maximum current density (Table 4).

**Table 4.** Two-way ANOVA statistical parameters for characterizing effects of sex and IHKA treatment on *I*_A_

Property	Sex	Treatment	Sex: treatment	Overall
*V*_1/2_ inactivation	***F*_(1, 55)_** = **4.065, *p* = 0.049**	*F*_(1, 55)_ = 0.023, *p* = 0.881	*F*_(1, 55)_ = 1.017, *p* = 0.318	*F*_(3, 55)_ = 1.538, *p* = 0.215
Inactivation slope factor	***F*_(1, 55)_ = 13.723, *p* = 0.00049**	*F*_(1, 55)_ = 1.162, *p* = 0.286	*F*_(1, 55)_ = 2.277, *p* = 0.137	***F*_(3, 55)_ = 5.973, *p* = 0.0013**
*V*_1/2_ activation	*F*_(1, 56)_ = 0.624, *p* = 0.433	*F*_(1, 56)_ = 1.018, *p* = 0.317	*F*_(1, 56)_ = 1.779, *p* = 0.188	*F*_(3, 56)_ = 1.070, *p* = 0.369
Activation slope factor	*F*_(1, 56)_ = 3.519, *p* = 0.066	*F*_(1, 56)_ = 1.418, *p* = 0.239	*F*_(1, 56)_ = 0.169, *p* = 0.683	*F*_(3, 56)_ = 1.493, *p* = 0.226
*I*_A_ max	*F*_(1, 55)_ = 3.526, *p* = 0.066	*F*_(1, 55)_ = 3.130, *p* = 0.082	*F*_(1, 55)_ = 1.100, *p* = 0.299	*F*_(1, 55)_ = 2.646, *p* = 0.058
*I*_A_ max density	***F*_(1, 55)_ = 6.034, *p* = 0.017**	*F*_(1, 55)_* = *3.389, *p* = 0.071	*F*_(1, 55)_* = *1.578, *p* = 0.214	***F*_(3, 55)_ = 3.734, *p* = 0.016**

Parameters shown in bold showed significant differences.

**Figure 7.**
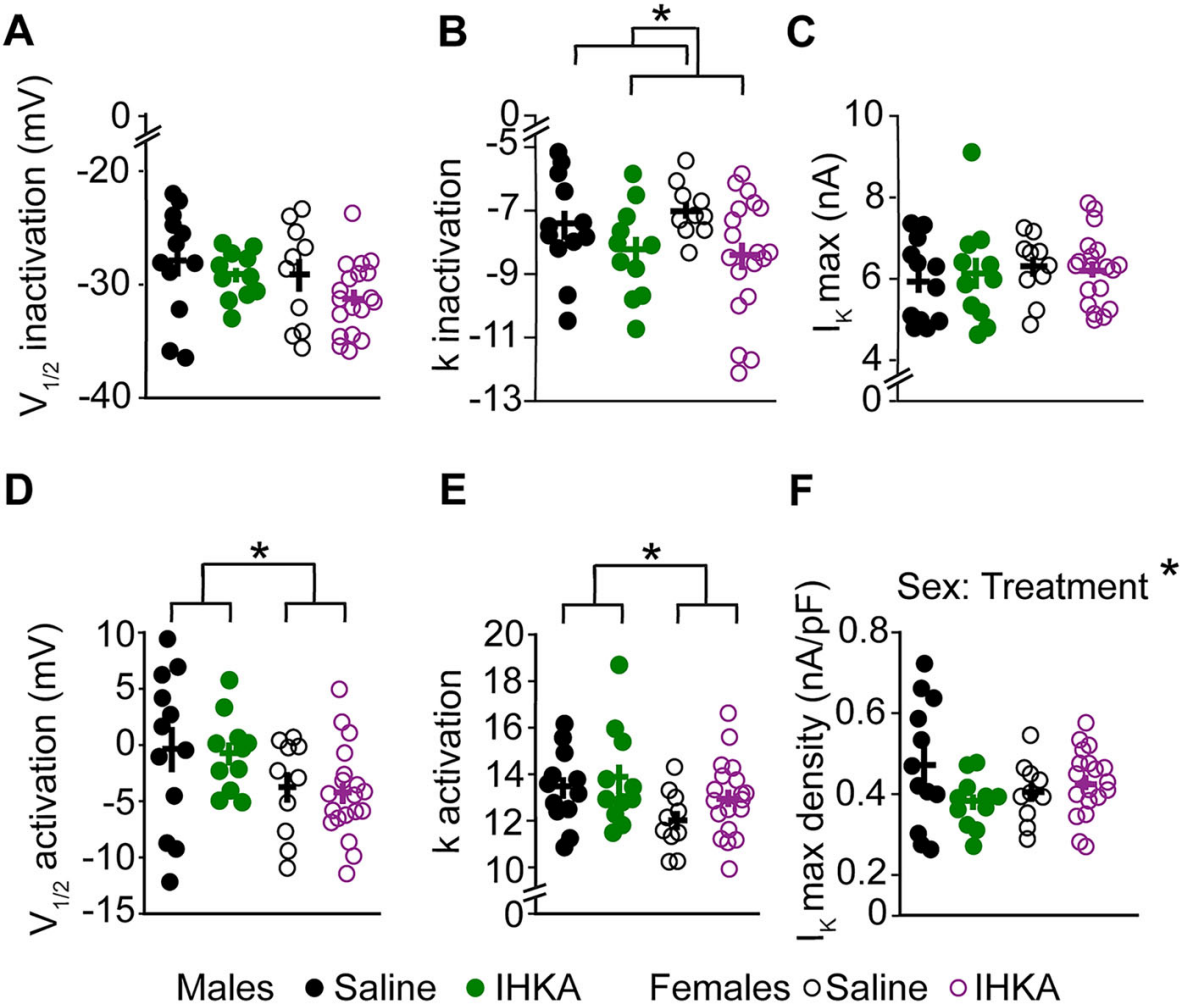
Effects of sex and IHKA status on the properties of *I*_K_. ***A***, *V*_1/2_ inactivation. ***B***, *k* inactivation slope factor; *p < 0.05 using two-way ANOVA. ***C***, Maximum current. ***D***, *V*_1/2_ activation; *p < 0.05 using two-way ANOVA. ***E***, *k* activation slope factor; *p < 0.05 using two-way ANOVA. ***F***, Maximum current density; *p < 0.05 using two-way ANOVA. Note that data from IHKA-regular and IHKA-long female mice are combined.

For the *V*_1/2_ inactivation of *I*_K_, no differences based on sex, treatment, or interaction were observed. For the inactivation slope factor, treatment showed a significant effect (*p* = 0.016) showing faster inactivation for IHKA-injected mice, whereas sex and the interaction between sex and treatment did not show any difference. A significant effect of sex was found for the *V*_1/2_ activation (*p* = 0.016) with males having a more depolarized *V*_1/2_ activation than females and activation slope factor of *I*_K_ (*p* = 0.026), showing faster activation for males. However, there was no effect based on treatment or interaction between sex and treatment. There were also no differences based on sex, treatment, or interaction for *I*_K_ maximum current, although for *I*_K_ maximum current density, the interaction between sex and treatment was significant (*p* = 0.044), indicating a combined effect on *I*_K_ maximum current density. However, neither sex nor treatment alone had an effect (Fig. 7). These findings suggest that certain ion channel properties, such as the inactivation slope factor and *V*_1/2_ activation of *I*_K_, are also influenced by sex or IHKA treatment but other properties such as *V*_1/2_ inactivation, *I*_K_ maximum current, and *I*_K_ maximum current density show limited significant differences, except for a notable interaction effect on *I*_K_ maximum current density (Table 5).

**Table 5.** Two-way ANOVA statistical parameters for characterizing effects of sex and IHKA treatment on *I*_K_

Property	Sex	Treatment	Sex: treatment	Overall
*V*_1/2_ inactivation	*F*_(1, 48)_ = 2.394, *p* = 0.128	*F*_(1, 48)_ = 2.346, *p* = 0.132	*F*_(1, 48)_ = 0.108, *p* = 0.744	*F*_(3, 48)_ = 2.055, *p* = 0.119
Inactivation slope factor	*F*_(1, 48)_ = 0.117, *p* = 0.734	***F*_(1, 48)_ = 6.213, *p* = 0.016**	*F*_(1, 48)_ = 0.519, *p* = 0.474	*F*_(3, 48)_ = 2.347, *p* = 0.084
*V*_1/2_ activation	***F*_(1, 48)_ = 6.242, *p* = 0.015**	*F*_(1, 48)_ = 0.116, *p* = 0.734	*F*_(1, 48)_ = 0.004, *p* = 0.949	*F*_(3, 48)_ = 2.344, *p* = 0.085
Activation slope factor	***F*_(1, 48)_ = 5.252, *p* = 0.026**	*F*_(1, 48)_ = 1.875, *p* = 0.177	*F*_(1, 48)_ = 0.377, *p* = 0.542	*F*_(3, 48)_ = 2.144, *p* = 0.107
*I*_K_ max	*F*_(1, 48)_ = 0.621, *p* = 0.43458	*F*_(1, 48)_ = 0.047, *p* = 0.829	*F*_(1, 48)_ = 0.204, *p* = 0.654	*F*_(3, 48)_ = 0.285, *p* = 0.836
*I*_K_ max density	*F*_(1, 48)_ = 0.123, *p* = 0.728	*F*_(1, 48)_ = 1.477, *p* = 0.230	***F*_(1, 48)_ = 4.259, *p* = 0.044**	*F*_(3, 48)_ = 1.838, *p* = 0.153

Parameters shown in bold showed significant differences.

## Discussion

People with TLE are at higher risk of developing reproductive endocrine comorbidities than the general population (Herzog, 2008). However, the mechanisms underlying this association are unknown. Previous studies have demonstrated increased GnRH neuron intrinsic excitability in IHKA mouse model of TLE in both males and females with/without estrous cycle disruption (Li et al., 2018). We used GnRH-GFP transgenic male and female mice to investigate the effects of epilepsy in the IHKA mouse model on the intrinsic excitability and voltage-gated K^+^ currents in GnRH neurons. We showed that IHKA males displayed increased GnRH neuron intrinsic excitability, increased *I*_A_ current density in the absence of other changes in *I*_A_ properties, and slower *I*_K_ recovery from inactivation. Furthermore, no changes in voltage-gated K^+^ currents were found in IHKA female mice, demonstrating an effect of sex on the response of voltage-gated K^+^ conductances in GnRH neurons to the challenge of epilepsy. Our studies also indicate sex-specific influences on certain ion channel properties, particularly the inactivation characteristics of *I*_A_, the maximum *I*_A_ current density, and the activation characteristics of *I*_K_, in IHKA mice that are not observed in controls.

A range of ionic currents have been implicated as contributing factors to epilepsy, with K^+^ currents playing a major role (Birnbaum et al., 2004; Timofeev et al., 2004). In the methylazoxymethanol model of cortical malformations and epilepsy, the disorganized pyramidal neurons in the hippocampus lack K_v_4.2 subunit, responsible for *I*_A_ currents, leading to hyperexcitable firing patterns (Castro et al., 2001; X. Chen et al., 2006). In TLE, decreased A-type K^+^ channel availability increases dendritic excitability of CA1 pyramidal neurons (Bernard et al., 2004). Mutations in K_v_4.2 are linked to neuronal excitability and reduced K^+^ current density, with variants reported in patients with TLE and autism with intractable seizures (Singh et al., 2006; Lee et al., 2014). Mutations in the KCNA1 (K_V_1.1) significantly reduce the amplitude of K^+^ currents by impacting the *I*_K_ currents, thereby increasing excitability and are linked to partial epilepsy (Spauschus et al., 1999; Zuberi et al., 1999). K_V_2.1 knock-out mice, with reduced *I*_K_ currents, show hyperexcitability and increased convulsant sensitivity (Speca et al., 2014). Variants of K_V_2.1 and K_V_1.6 are associated with epileptic encephalopathy and infantile-onset epilepsy with neurodevelopmental disorders (Torkamani et al., 2014; Thiffault et al., 2015; Salpietro et al., 2023).

Other studies have demonstrated changes in voltage-gated *I*_A_ and *I*_K_ current properties in neuronal populations outside the seizure foci. For example, decreased *I*_A_ current amplitude in the nucleus tractus solitarius neurons potentially contributes to sudden unexpected death in epilepsy in a mouse model of acquired TLE (Derera et al., 2019). With respect to hypothalamic neurons, changes in *I*_A_ and *I*_K_ currents occur not only in epilepsy but in various other conditions. Prorenin inhibits *I*_A_, alters action potential waveform, and increases excitability in hypothalamic vasopressin neurosecretory neurons (Pitra and Stern, 2017). Similarly, angiotensin II inhibits *I*_A_ in paraventricular nucleus (PVN) neurons, contributing to increased excitability. Diminished expression of K_V_4.3, which mediates *I*_A_, increases neuronal excitability in PVN neurons in hypertensive rats (Sonner et al., 2011). Leptin decreases neuronal excitability in agouti-related peptide/neuropeptide Y coexpressing neurons by increasing *I*_K_; this effect is impaired in diet-induced obese mice, leading to decreased *I*_K_ and increased excitability (Baver et al., 2014). These findings link *I*_A_ and *I*_K_ currents to increased excitability in hypothalamic neurons. Therefore, we were interested in characterizing these currents in GnRH neurons to understand their role in neuronal excitability in a mouse model of TLE.

The present studies show a significant increase in the intrinsic excitability of GnRH neurons in both male and female GnRH-GFP transgenic mice following IHKA injections. These findings align with previous studies that showed increased excitability in GnRH neurons from GnRH-Cre:Ai9 mice upon IHKA treatment (Li et al., 2018). The increased intrinsic excitability seen in both IHKA males and females indicates enhanced neuronal excitability in IHKA mice, irrespective of sex. Furthermore, the treatment effect on the evoked firing rate did not differ significantly between male and female mice, suggesting a generalized response to IHKA across sexes. Moreover, this phenotype is well preserved across different mouse strains, indicating a robust and consistent alteration in GnRH neuron excitability in the IHKA model.

Despite the increased intrinsic excitability seen in GnRH neurons, the present results do not indicate differences in the voltage dependence of inactivation and activation of *I*_A_ in IHKA male mice. These results suggest that the increased intrinsic excitability in GnRH neurons is not mediated by changes in *I*_A_ currents. These findings are intriguing given that *I*_A_ currents are known to play a crucial role in regulating neuronal excitability and action potential firing. It is possible that changes in *I*_A_ currents are not uniformly observed across all cell types in epilepsy or across all types of epilepsy. For example, genetic mutations that affect ion channel genes lead to specific channelopathies where A-type currents are either increased or decreased. Mutations in the KCNA1 gene alter K_V_1.1 function and subsequently affect neuronal excitability (Spauschus et al., 1999). By contrast, no chronic changes in *I*_A_ were observed in hippocampal dentate granule cells from rats in the pilocarpine poststatus epilepticus model of TLE (Rüschenschmidt et al., 2006). The present study shows a significant increase in *I*_A_ maximum current density in GnRH neurons in IHKA male mice, which would be expected to cause a decrease in neuronal excitability. However, this finding appears to be in contradiction with the hyperexcitability phenotype seen in these mice. Moreover, the unchanged capacitance and *I*_A_ current amplitude indicate that the increase in *I*_A_ current density is not due to changes in cell size but might instead reflect changes in channel expression or function that do not translate into a major physiological effect. This suggests that the increase in *I*_A_ current density in IHKA male mice, in the absence of changes in other parameters, may not be sufficient to counterbalance the overall hyperexcitability seen in IHKA male mice.

In contrast to the unchanged *I*_A_ currents, the present results indicate that GnRH neurons from IHKA males exhibited slower *I*_K_ recovery from inactivation compared with respective saline controls. A slower recovery upon IHKA treatment would imply changes in the functional properties of these channels, possibly through posttranslational modifications such as dephosphorylation or oxidation of K_V_2.1. A similar mechanism was seen in rats subjected to KA-induced status epilepticus (Misonou et al., 2004, 2005), with marked dephosphorylation of K_V_2.1 in cortical and hippocampal pyramidal neurons and significant delay in the recovery from inactivation of *I*_K_. Based on their phosphorylation state, these channels function as dynamic resistors in the somatic membrane, leading to increased or decreased neuronal excitability. By contrast, the voltage dependence of inactivation and activation and time course of inactivation of *I*_K_ were not altered in GnRH neurons from IHKA males. Overall, these results indicate that specific kinetic properties of *I*_K_ in GnRH neurons are modified in IHKA males, thereby likely influencing cellular excitability. Further investigations into the molecular mechanisms underlying changes in the recovery kinetics of *I*_K_ currents in GnRH neurons in IHKA mice could provide valuable insights into the specific role of *I*_K_ currents in increasing excitability in epilepsy.

The present results demonstrate a lack of differences in the voltage dependence of inactivation and activation of both *I*_A_ and *I*_K_ currents in IHKA female mice. In GnRH neurons, alterations in excitability can affect hormone release and overall reproductive health. Since *I*_A_ and *I*_K_ currents do not appear to underlie increased excitability in females, other mechanisms are likely responsible. GnRH neurons exhibit a multiplicity of ionic conductances (Norberg et al., 2013) which determine the pattern of neuronal firing, excitability, and action potential waveforms, including TTX-sensitive voltage–gated sodium channels (Wang et al., 2010). TTX-sensitive sodium channels, primarily Na_V_1.1, Na_V_1.2, Na_V_1.3, and Na_V_1.6, play essential roles in the generation and propagation of action potentials in neurons and are critically involved in the pathophysiology of epilepsy. GnRH neurons also express prominent high-voltage–activated and small low-voltage–activated calcium currents (Nunemaker et al., 2003; Sun et al., 2010). Lastly, other K^+^ currents in GnRH neurons, such as small- and large-conductance calcium–activated currents, M-type currents, and ATP-sensitive K_ATP_ currents (Bosch et al., 2002; Zhang et al., 2007; Hiraizumi et al., 2008; Liu and Herbison, 2008; Xu et al., 2008), may be fruitful avenues for future investigation.

The present findings indicate influences of sex on properties of *I*_A_ and *I*_K_. Significant changes were observed in *V*_1/2_ inactivation, the inactivation slope factor *k*, and maximum current density of *I*_A_. These results suggest that the properties of voltage-dependent inactivation of *I*_A_ channels in GnRH neurons display distinct sensitivities to epilepsy in male vs female mice. Additionally, changes in slope factor suggest variations in the kinetics of *I*_A_ channel inactivation. Moreover, we found differences in the maximum current density, indicating variations in the overall magnitude of *I*_A_ currents based on sex. These findings collectively suggest that inherent differences between male and female mice influence the properties of *I*_A_ currents. Furthermore, the interaction between sex and IHKA treatment significantly affected *I*_K_ maximum current density. This finding suggests that the combined effect of sex and IHKA treatment modulates these specific properties of *I*_K_ currents. Control males and females did not exhibit differences in the properties of *I*_A_ and *I*_K_. This result is consistent with previous findings that showed no differences in these properties in GnRH neurons recorded from gonad-intact males and females (DeFazio and Moenter, 2021). These distinct sensitivities to parameters without changes in the voltage dependence of inactivation and activation of *I*_A_ and *I*_K_ likely result from a complex interplay of hormonal, genetic, molecular, and cellular factors. Further research into these mechanisms can provide deeper insights into sex-specific modulation of these currents in GnRH neurons in IHKA mice.

The findings of this study provide several implications for understanding epilepsy-associated changes in GnRH neuron function. The lack of change in *I*_A_ voltage and time dependence in IHKA males suggests that *I*_A_ currents do not mediate increased GnRH neuron excitability. However, changes in *I*_K_ recovery from inactivation in IHKA males highlight *I*_K_ channels as exhibiting epilepsy-associated changes in biophysical properties. GnRH neurons from IHKA females do not exhibit changes in *I*_A_ and *I*_K_, suggesting sex-specific responses to epilepsy at the level of ionic conductances in GnRH neurons. Lastly, sex differences observed in IHKA but not control mice indicate that epilepsy may exacerbate certain sex differences in GnRH neuron K^+^ current properties. Overall, this study contributes to a deeper understanding of the cellular mechanisms underlying increased excitability in GnRH neurons in the IHKA mouse model of epilepsy.

## Synthesis

Reviewing Editor: Deanna Smith, University of South Carolina Decisions are customarily a result of the Reviewing Editor and the peer reviewers coming together and discussing their recommendations until a consensus is reached. When revisions are invited, a fact-based synthesis statement explaining their decision and outlining what is needed to prepare a revision will be listed below. The following reviewer(s) agreed to reveal their identity: Richard Piet.

The reviewers and I agree that the manuscript confirms that temporal lobe epilepsy affects the excitability of gonadotropin-releasing hormone (GnRH) neurons in a mouse model. The electrophysiology is well-described, the results are for the most part well-explained and well-illustrated, with high quality sample traces. The findings are interesting and the manuscript is overall well-written. The experiments appear to be rigorously conducted and well-controlled (TLE epilepsy is confirmed behaviorally in all mice, and using histology to assess hippocampal damage in cases where behavior was ambiguous).

The demonstration that potassium conductances (IA and IK currents) do not drive the hyperexcitability of GnRH neurons in temporal lobe epilepsy is indicated, but there are some concerns that Figure 2 shows a significant difference in the peak IA current density, with larger densities in GnRH neurons from male IHKA mice. The authors note this difference but suggest it is "modest" (lines 368-369). However, the observed 30-40% difference in peak current density is possibly more than a modest one. Although the peak amplitude is not significantly different, the current density is a better index as it accounts for potential variability in cell size. The authors should take this difference into account in the interpretation of their findings.

Below are some other suggestions to improve the manuscript:

1. Statistics should be revised using a nested, mixed, or marginal model. Statistics run on cells is common but it is a violation of independence for ANOVA. It would be far better to employ a mixed effects model with animals as a random effect or an ANOVA with cell nested within animal.

2. The description of statistical tests and results is at times difficult to follow: Which test was used and when was not always clear; for example, lines 301-302 indicate that Tukey HSD tests were used for within male group comparisons, but the Figure 2 legend indicates that this was done with independent t-tests. p values are sometimes given in the text, sometimes not. Results of two-way ANOVA analyses could be clearer especially in the tables. For example, what are the p values given in tables 2 and 3?

3. in Figure 1, the statistical analysis performed in B is not described in the main text, while the legend indicates that it was done using a two-way ANOVA but there are only two groups (AUC saline vs AUC KA). For panel D, the main text describes the use of a one-way ANOVA but the legend states two-way ANOVA.

4. Figure 6 is hard to read and to reconcile with tables 4 and 5 and the text - which panels relate to IA or to IK is not immediately obvious. It is suggested that the figure be split into two, one for IA and one for IK, displaying all 5 parameters for each current.

5. Lines 115-116 are unclear to the reviewer, please revise.

6. Lines 148-149: zeitgeber time should be relative to the time lights are turned on.

7. Line 221: please revise, it is the prepulse that inactivates the current.

8. Lines 289: "dividing"

9. Lines 289-296: it is stated that normalized peak currents were divided by the driving force at each voltage step. Thus, these values should be referred to as conductances, not currents, in the text and in figures 2B-D, 3C-E, 4A-C and 5A-C.

10. Line 338: there is a slight discrepancy between the p value at the end of the sentence and that reported in table 1.

## Author Response

Synthesis of Reviews:

Synthesis Statement for Author (Required):

The manuscript is much improved. However, please address an issue regarding the increased IA current density seen in IHKA male mice. While the added discussion (lines 512-520) is appreciated, the authors may also revise the abstract (line 13), results section (subheading line 358) and discussion (line 456) to account for the difference in IA seen in males.

We have made the requested modifications to the abstract, results, and discussion to note the change in IA current density observed in IHKA male mice.
